# The safety and efficacy of transcranial direct current stimulation as add-on therapy to fluoxetine in obsessive-compulsive disorder: a randomized, double-blind, sham-controlled, clinical trial

**DOI:** 10.1186/s12888-020-02979-1

**Published:** 2020-11-30

**Authors:** Sadegh Yoosefee, Man Amanat, Mona Salehi, Seyed Vahid Mousavi, Jamshid Behzadmanesh, Victoria Safary, Ali Yoonesi, Bahman Salehi

**Affiliations:** 1 Neurosciences Research Center, Qom University of Medical Sciences, Qom, Iran; 2 Spiritual Health Research Center, Qom University of Medical Sciences, Qom, Iran; 3 Faculty of Medicine, Students’ Scientific Research Center, Tehran University of Medical Sciences, Tehran, Iran; 4 NeuroImmunology Research Association (NIRA), Universal Scientific Education and Research Network (USERN), Tehran, Iran; 5 Department of Psychiatry, Arak University of Medical Sciences, Arak, Iran; 6 Department of Neurosciences, School of Advanced Technologies in Medicine, Tehran University of Medical Sciences, Tehran, Iran

**Keywords:** Obsessive-compulsive disorder, Transcranial direct current stimulation, Fluoxetine, Yale-Brown obsessive-compulsive scale, Anxiety

## Abstract

**Background:**

Obsessive-compulsive disorder (OCD) is an anxiety disorder that causes impairment in daily activities. This study aimed to assess the safety and efficacy of transcranial direct current stimulation (tDCS) as adjunctive therapy with fluoxetine in individuals diagnosed with moderate to severe OCD.

**Methods:**

This is a randomized, double-blind sham-controlled trial. Individuals with OCD who had baseline Yale-Brown obsessive-compulsive scale (Y-BOCS) of > 15 were enrolled. Eligible cases were randomly assigned in 1:1 ratio to receive either 20-min-period of stimulation with tDCS and fluoxetine (experimental arm) or fluoxetine only (sham control arm). The anodal electrode of tDCS was placed over the left dorsolateral prefrontal cortex (Fp3) and the cathodal electrode was placed over the right orbitofrontal cortex (F8). Two mA electrical stimulation with the tDCS was used for 20 min in individuals of experimental group. In the control group, electrodes were placed and stimulation was administered for 30 s to induce the same skin sensation as in experimental group. This procedure was performed three times per week for 8 weeks. Y-BOCS test was assessed at baseline, week 4 (after 12th stimulation), week 8 (after 24th stimulation), and 1 month after the last stimulation. The primary endpoints were the mean changes in Y-BOCS total score from baseline to the last visit. The secondary endpoints were the mean changes in obsession and compulsion sub-scores from baseline to the last visit. Adverse events were also assessed. Mixed design repeated measures analysis of variance assessed the endpoints.

**Results:**

Sixty individuals (30 in each group) were participated. All individuals in control group and 28 cases in experimental arm completed the trial. The mean Y-BOCS (F_(1.85)_ = 30.83; *P* < 0.001), OCD obsession (F_(2.23)_ = 25.01; *P* < 0.001), and compulsion (F_(2.06)_ = 10.81; *P* < 0.001) scores decreased significantly during the study. No statistical differences were, however, detected between experimental and control groups (*P* > 0.05). The tDCS was well tolerated and no major adverse events were reported.

**Conclusion:**

This study showed that among individuals with moderate to severe OCD, there was no significant difference regarding OC symptoms between cases used tDCS as adjunctive therapy with fluoxetine and individuals used fluoxetine only.

**Trial registration:**

IRCT2017030632904N1. Registered 14 July 2017, http://irct.ir/user/trial/44193/view

## Background

Obsessive-compulsive disorder (OCD) is a debilitating condition characterized by recurring, unwanted thoughts and ideas (*obsessions*) and behaviors (*compulsions*). The global prevalence of OCD is about 1 to 2% [1, 2] and is more frequent among females [2]. Studies showed that OCD is associated with decreased levels of serotonin [3–5]. Selective serotonin re-uptake inhibitors (SSRIs) are, therefore, often used as the first-line treatment in OCD [6]. In non-responders, serotonin-norepinephrine re-uptake inhibitors (e.g. venlafaxine), tricyclic antidepressants (e.g. clomipramine), and atypical antipsychotics (e.g. aripiprazole) are suggested for therapy. About 40 to 60% of people with OCD achieve remission with these pharmacotherapeutic options [7]. Cognitive-behavioral therapy (CBT), is also useful in individuals with OCD. Evidence showed that CBT is effective in more than one-third of people with OCD but they are at risk of relapse [8]. When mono-therapy is not effective, other approaches including “combining”, “augmenting”, and “switching” strategies can be used but they may not always provide adequate relief and may cause significant adverse events [9, 10]. New therapeutic options are, therefore, needed.

Emerging evidence suggested that OCD was associated with impaired function in the fronto-striatal-thalamic-cortical loop circuits including the dorsolateral prefrontal cortex (DLPFC), the orbitofrontal cortex, medial prefrontal cortex (e.g. anterior cyngulate gyrus), supplementary motor area, and the basal ganglia [11, 12]. A neuromodulatory method known as transcranial direct current stimulation (tDCS) showed promising results in several neuropsychiatric disorders [13–16]. Direct electrical stimulation of the brain over the skull is a non-invasive and safe method with minor side-effects. In tDCS, weak direct current (1 to 2 mA) is applied to the scalp of subjects [17] that flows from the anode to the cathode with a fraction of the current entering the brain. The tDCS can cause more or fewer neurons to fire by alternating the excitability of neurons and shifting the membrane potential of superficial neurons to depolarize or hyperpolarize [18]. Some earlier uncontrolled and small sample-sized studies have shown that tDCS may improve symptoms of OCD [19–21]. This randomized clinical trial (RCT) was designed to evaluate the safety and clinical efficacy of tDCS as add-on therapy to fluoxetine in individuals with moderate to severe OCD. We hypothesized that improvements in obsessive compulsive symptoms by using tDCS as an adjunctive therapy with fluoxetine were not worse than using fluoxetine only (non-inferiority).

## Methods

### Study design and sample

This was a randomized double-blind sham-controlled trial conducted in Amir Kabir Hospital, affiliated to Arak University of Medical Sciences, Iran. Individuals aged 18 to 60 years with recent diagnosis of moderate to severe OCD based on DSM-V criteria [22] were included. To assess the severity of OCD, Yale-Brown obsessive compulsive scale (Y-BOCS) was used and cases with the baseline score above 15 were enrolled. Individuals with the history of any physical or psychiatric conditions but OCD or the history of substance use were excluded. Other exclusion criteria were taking monoamine oxidase inhibitors or antipsychotics for any reason; concurrent use of CBT; changes in the treatment regimen of OCD in the last 6 months; electroconvulsive therapy (ECT) during the last 6 months; pregnancy; no prior therapeutic response to SSRI, and the history of adverse events with SSRI use.

The trial was conducted according to the original protocol. The protocol of this study was reviewed and approved by the local ethics committee (IR.ARAKMU.REC.1395.9). Each subject received a full description of the study. Written informed consent was obtained from all subjects. The study was conducted with adherence to CONSORT guidelines and was registered prospectively with Iranian Registry of Clinical Trials, IRCT.ir; number: IRCT2017030632904N1.

### Randomization and masking

Included participants were randomly assigned in 1:1 ratio via an interactive web response system to receive either tDCS and fluoxetine or fluoxetine only. Permuted block randomization method (block size = 4) was used for group allocation. Participants and investigators were blinded during the study. Two individuals (a nurse and a medical doctor) were responsible to perform tDCS on participants. They were trained in the procedure of tDCS and were informed about the aim of the study. They were the only individuals who were not masked during the trial but they had no information regarding the clinical data of participants. They had no contacts with investigators and were monitored during the procedure so they did not reveal any information to participants. The responsible statistician was not aware of the clinical characteristics of subjects. All cases were connected to the tDCS via electrodes and electric current was administered to the sham control group for 30 s so the same sensation as in experimental group (tingling of skin) was induced. The electrodes were remained around the head of these cases for further 20 min without electrical stimulation.

### tDCS procedure

Direct electrical stimulation was started with the tDCS device (manufactured by Medina Teb, Iran). Two electrodes (size: 7 cm × 5 cm) referenced as anode and cathode were used to deliver stimulation and were held around the head by non-conducting bandage. To minimize the noise pollution, the doors of recording room were closed during the experiment.

The anodal electrode generally increases (depolarize neurons) and the cathodal electrode decreases (hyperpolarize neurons) the cortical excitability [18]. Anodal stimulation of left DLPFC was shown to decrease the hyperactivity of Delta and Theta bands and normalize the electroencephalography (EEG) pattern of cases with OCD [23]. The anode electrode of tDCS device was, therefore, placed on the left DLPFC equivalent to Fp3 point of the international 10–20 EEG system. The cathode electrode was placed on the right lateral aspect of orbit (F8 point of the 10–20 EEG system) to target right orbitofrontal cortex (OFC). A current of 2 mA was set for stimulation. Prior to any electric stimulation, the skin surface was examined to assure the absence of burns, scratches, redness, pain, and inflammation. The electrodes were, then, covered in sponges soaked with 0.9% saline solution to prevent pain and burns during stimulation. Upon the patient’s announcement of readiness, the electric current was exerted in a 20 min-period in experimental group. Additional ramp up (15 s) and ramp down (15 s) stimulation was administered at the beginning and end of the session; respectively. The current of 2 mA was used for 30 s in sham control arm. This procedure was performed three times per week for 8 weeks. All patients received 20 mg of fluoxetine twice each day. The dose of fluoxetine was not changed during the study period.

### Yale-Brown obsessive compulsive scale

The scale was developed by Goodman et al. [24], and consists of 10 items (5 items to assess the obsessive thoughts and 5 items for compulsive behaviors). Each item can be scored on a 5-point scale (0–4). Severity, frequency, and duration of symptoms as well as trying to resist the symptoms and the affect of symptoms to interfere with everyday life of cases will be questioned in this test [24]. People with the diagnosis of OCD can be classified based on the total score of the test:

8 to 15: mild; 16 to 23: moderate; 24 to 31: severe; 32 to 40: extreme.

The study on 40 patients showed that the reliability of Yale-Brown obsessive compulsive scale (Y-BOCS) was 0.98 and the internal consistency coefficient (alpha coefficient) was 0.89 [24]. The study on 140 cases with OCD indicated that the Farsi version of Y-BOCS had also satisfactory reliability and validity (internal consistency score of 0.97 for symptom checklist; internal consistency score of 0.95 for severity scale; and test-retest reliability of 0.99) [25].

### Clinical assessments

At baseline, Demographic and clinical data were obtained by interviewing individuals. Clinical symptoms and Y-BOCS scores were measured at baseline and during follow-ups at week 4 (after 12th session), week 8 (after 24th session), and 1 month after the last session. The primary endpoints were the mean changes in Y-BOCS total score from baseline to the last follow-up visit. The secondary endpoints were the mean changes in Y-BOCS obsession and compulsion sub-scores from baseline to the last follow-up visit. Adverse events were also assessed. During the follow-up visits we asked the participants to report any side effects. A phone number was also provided so the cases could ask their questions or report any side effects as soon as possible. Participants were requested to visit the emergency department if a major complication happened. A list of common adverse events of SSRI and tDCS was also provided so the cases were aware of those conditions. The interview and procedures were all performed in a calm and stress-free situation for the examiner and participants.

### Statistical data analysis

When this study was designed in 2017, no similar placebo-controlled study was available. Therefore, we assumed effect size as 0.15 (F = 0.15) to calculate the sample size (statistical test: repeated measures, within-between interaction). Considering α (the probability of type I error) as 0.05 and β (the probability of type II error) as 0.20, the required total sample size was 60 (30 participants per group). G*Power software was used for sample size calculation.

Data were analyzed by IBM SPSS Software, version 25.0 (SPSS Inc., Chicago, IL). Descriptive statistics (mean and SD, or number and %) were used to express demographic and clinical data. The normality of data were examined using Kolmogorov-Smirnov test. Baseline characteristics were compared between groups using chi-square tests for categorical variables and independent sample t-tests for numeric variables. Mixed design repeated measures analysis of variance (ANOVA) with terms for group, time, and time*group interaction was used to evaluate changes in outcome measures during the study period. Cohen’s d test with 95% confidence interval (CI) were used to measure the effect sizes that were classified as small (d: 0 to 0.20), medium (d: 0.20 to 0.50), and large (d > 0.50). Two-tailed *p*-values< 0.05 were considered statistically significant.

## Results

### Baseline characteristics

**Fig. 1:**
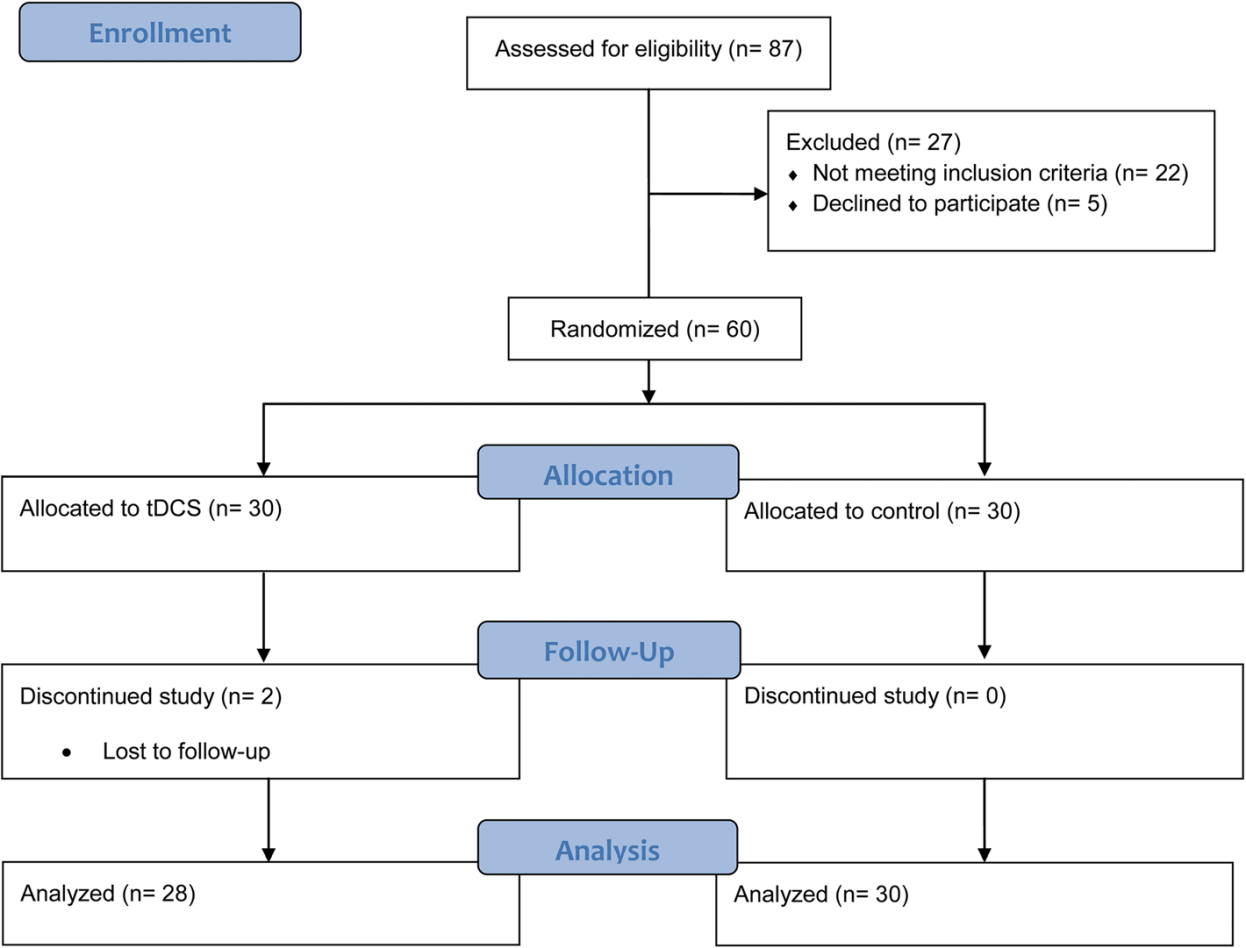
CONSORT Flow diagram

The trial started on March 21, 2017, when the recruitment of participants with OCD was begun. The double-blind treatment was given from May 2, 2017, when the first individual was assigned to July 23, 2018. A total of 60 individuals were randomly assigned to tDCS (*n* = 30) and sham control (*n* = 30) groups. All participants completed 24 sessions of the allocated treatment. Two individuals in tDCS arm were lost for the last follow-up visit. None of these missed participants reported adverse events. Overall, 28 individuals in the tDCS group and all participants in the sham control arm completed the study (Fig. 1). The baseline clinical and demographic characteristics of participants are described in Table 1. No statistical differences were observed between two arms of study regarding baseline characteristics.

**Table 1:** Baseline clinical and socio-demographical characteristics of the recruited patients with OCD

	tDCS (n = 30)	Control (n = 30)	P-value
Age, year [mean ± SD]	38.6 ± 12.6	35.9 ± 11.5	0.395
Gender, male [n (%)]	4 (13.3)	7 (23.3)	0.506
Marital status [n (%)]			0.379
Single	10 (33.3)	15 (50.0)	
Married	19 (63.3)	13 (43.3)	
Divorced	1 (3.3)	2 (6.7)	
Education [n (%)]			0.999
Under diploma	10 (33.3)	10 (33.3)	
Diploma	13 (43.3)	14 (46.7)	
Bachelor’s	7 (23.3)	6 (20.0)	
Occupation [n (%)]			0.312
Housekeeper	19 (63.3)	13 (43.3)	
Employed	3 (10.0)	6 (20.0)	
Unemployed	8 (26.7)	11 (36.7)	
Smoking [n (%)]	2 (6.7)	0 (0.0)	0.492
Family history of psychiatric illness [n (%)]			0.7
Depression	1 (3.3)	3 (10.0)	
ADHD	1 (3.3)	0 (0.0)	
Bipolar disorder	2 (6.7)	1 (3.3)	
OCD	11 (36.7)	13 (43.3)	
Socioeconomic status [n (%)]			0.205
Very low	4 (15.4)	6 (20.7)	
Low	6 (23.1)	9 (31.0)	
Moderate	7 (26.9)	11 (37.9)	
High	9 (34.6)	3 (10.3)	
Hospitalization [n (%)]	6 (20.0)	7 (23.3)	0.754

*ADHD* attention deficit hyperactivity disorder; *OCD* obsessive–compulsive disorder

### Clinical outcomes

**Fig. 2:**
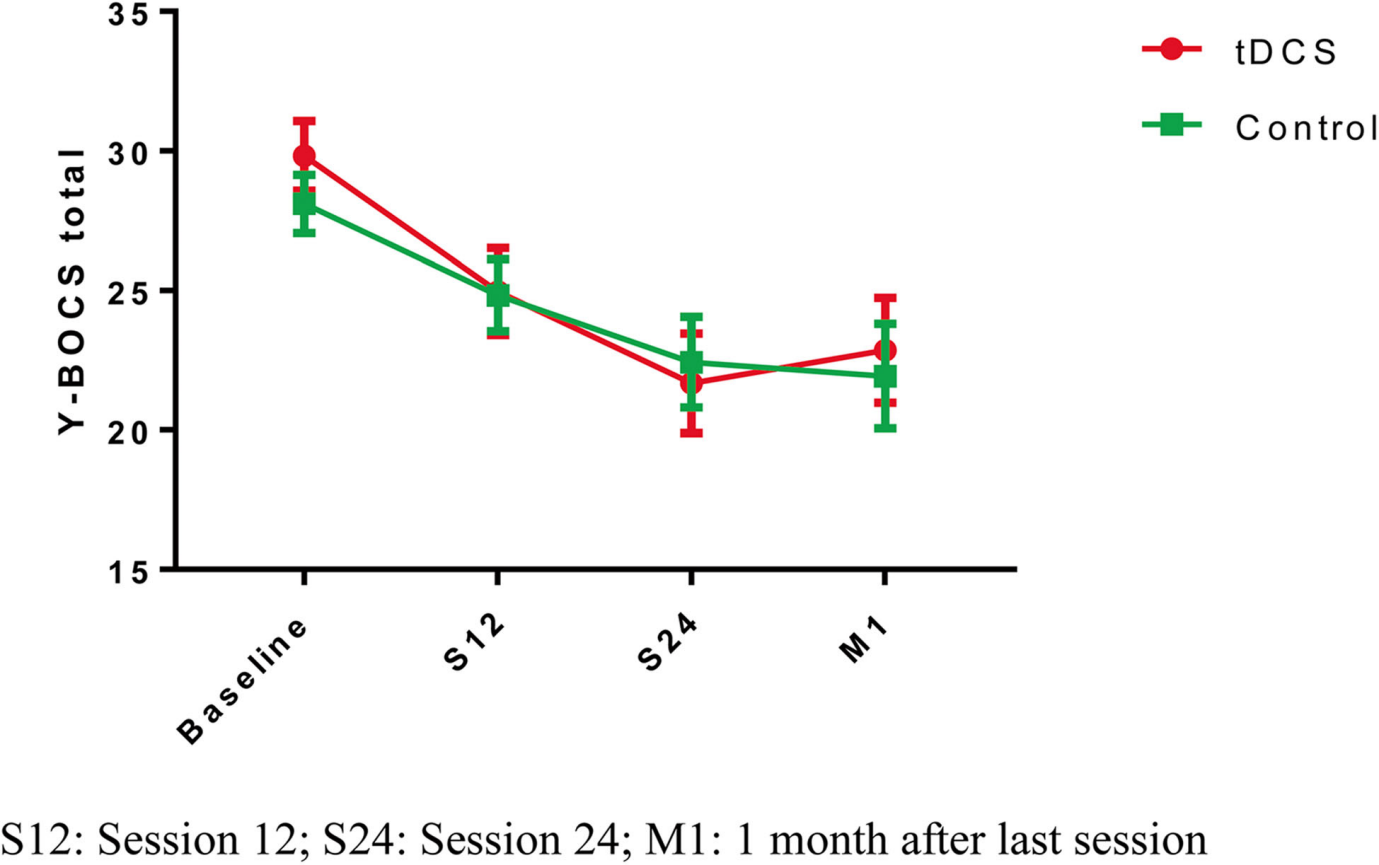
Y-BOCS score changes during the study period

Clinical measures at each study point are summarized in Table 2. The mixed design repeated-measures ANOVA for the total Y-BOCS score revealed a significant time effect (F_(1.85)_ = 30.83; *P* < 0.001) but no significant group effect (F_(1.00)_ = 0.07; *P* = 0.799) or interaction between time and group effect were observed (F_(1.85)_ = 0.86; *P* = 0.420) (Fig. 2). The Cohen’s d test for the Y-BOCS means at the last follow-up visit showed d: 0.079 with 95%CI: − 0.446 to 0.604.

**Table 2:** Clinical measures

	tDCS (*n* = 28)				Control (n = 30)				ANOVA (time)	ANOVA (time*group)	ANOVA (group)
	Baseline	S12	S24	M1	Baseline	S12	S24	M1			
Y-BOCS total	29.8 ± 6.6	25.0 ± 8.2	21.7 ± 9.5	22.9 ± 10.0	28.1 ± 5.7	24.8 ± 7.1	22.4 ± 8.9	21.9 ± 10.3	F_(1.85)_ = 30.83; *P* < 0.001	F_(1.85)_ = 0.86; *P* = 0.420	F_(1.00)_ = 0.07; *P* = 0.799
Y-BOCS obsession	15.2 ± 3.7	14.1 ± 3.5	11.1 ± 4.6	11.5 ± 5.0	14.1 ± 3.5	12.3 ± 4.4	11.1 ± 4.5	11.3 ± 5.4	F_(2.23)_ = 25.01; *P* < 0.001	F_(1.85)_ = 0.68; *P* = 0.523	F_(1.00)_ = 0.10; *P* = 0.748
Y-BOCS compulsion	14.3 ± 3.7	12.5 ± 4.8	11.7 ± 8.7	11.4 ± 5.3	14.0 ± 3.1	12.5 ± 3.7	11.3 ± 5.0	10.6 ± 5.5	F_(2.06)_ = 10.81; *P* < 0.001	F_(2.06)_ = 0.147; *P* = 0.869	F_(1.00)_ = 0.10; *P* = 0.748

*Y*-*BOCS* Yale–Brown Obsessive and Compulsive Scale; *S12* session 12; *S24* session 24; *M1* 1 month after the last session

Our study also showed that Y-BOCS obsession and compulsion scores decreased significantly during the study period (F_(2.23)_ = 25.01; *P* < 0.001 and F_(2.06)_ = 10.81; *P* < 0.001, respectively). No significant time*group interaction was, however, detected for Y-BOCS obsession (F_(1.85)_ = 0.68; *P* = 0.523) and compulsion (F_(2.06)_ = 0.147; *P* = 0.869) scores. The Cohen’s d test at the last follow-up visit showed d: 0.029 with 95%CI: − 0.496 to 0.555 for obsession mean scores and d: 0.119 with 95%CI: − 0.405 to 0.644 for compulsion mean scores.

Regarding safety endpoint, tDCS stimulation was well tolerated and no serious adverse events were reported. No cases left the study due to adverse events. Eight individuals in the tDCS group experienced minor adverse events (redness: 2 and irritation: 6). No adverse event occurred in the control group.

## Discussion

Our trial showed that individuals with moderate to severe OCD in the experimental group (tDCS stimulation + fluoxetine) had about 23% improvements in OC symptoms using Y-BOCS scale. Participants in sham controlled arm had about 22% improvements in symptoms. There was no statistical significant difference between two groups.

The lack of significant difference between groups might be partialy explained by tDCS parameters used in this trial. The most appropriate tDCS protocol regarding the electrode placement and the current delivered via electrodes are not well-established. The higher current dosage and longer sessions that were used in other psychiatric disorders might improve OC symptoms significantly [26]. Anodal electrode was placed over DLPFC in the present study. It was reported that DLPFC (Brodmann areas: 9 and 46) was associated with executive functions including working memory, selective attention, and maintaining or shifting sets in response to changing task demands [27]. The hypoactivation of DLPFC was observed in individuals with OCD [28]. Study on deep brain stimulation of cases with refractory OCD reported that DLPFC activation was associated with excellent clinical effects [29]. The cathodal electrode was placed over OFC in this trial. This cortical area was found to be hyperactive in most individuals with OCD [30] and successful treatments could normalize its function [31].

Different case-report studies assessed the efficacy of tDCS stimulation on individuals with OCD (Table 3). The results of these studies were inconsistent with each other. One study with one OCD case showed that tDCS stimulation was not associated with significant improvements in symptoms [32]. Another study with two participants indicated that one case had over 15% improvements with tDCS stimulation but the second individual had no statistical significant improvement [33]. Over 20 to 60% decrease in symptoms was observed in other studies [34–39]. We also found 3 non-randomized open label trials with small sample sizes (Table 3). These studies showed that tDCS was effective to improve OC symptoms compared to baseline [20, 40, 41].

**Table 3:** Studies investigated the efficacy of tDCS on individuals with OCD

Author, year	Study design	Anode and cathode position	Results
Volpato et al., 2013 [32]	Case report One 35 year-old-male	Anode: neck; Cathode: F3 2 mA, 20 min daily for 10 days	• Not effective on OC symptoms • Significant effect on depression and anxiety
Silva et al., 2016 [33]	Case report Two 31 and 37 year-old-males	Anode: left deltoid muscle; Cathode: pre-SMA 2 mA, 30 min daily for 20 days	• One participant had no improvement in Y-BOCS, depression, and anxiety • One participant had 55% reduction in Y-BOCS and 50% reduction in depression and anxiety symptoms
Mondino et al., 2015 [34]	Case report One 52 year-old-female	Anode: right occipital cortex; Cathode: FP1 2 mA, 20 min twice a day for 5 days	• 26% reduction in Y-BOCS
Narayanaswamy et al., 2015 [35]	Case report One 39 year-old-female and one 24 year-old-male	Anode: left pre-SMA; Cathode: right supra-orbital area 2 mA, 20 min twice a day for 10 days	• 46.7% reduction in Y-BOCS of male • 52% reduction in Y-BOCS of female
D’Urso et al., 2016 [36]	Case report One 33 year-old-female	Active: pre-SMA; Reference: right deltoid 2 mA, 20 min daily for 20 days	• 11% worsening in Y-BOCS after anodal sessions • 30% reduction in Y-BOCS after cathodal sessions
Alizadeh Goradel et al., 2016 [37]	Case report One 23 year-old-female	Anode: O2; Cathode: left OFC 2 mA, 20 min daily for 10 days	• 64% reduction in Y-BOCS; 87% reduction in depression; and 100% reduction in anxiety symptoms
Palm et al., 2017 [38]	Case report One 31 year-old-male	Anode: F3; Cathode: F4 2 mA, 30 min twice a day with total 20 stimulations in 2 weeks	• 22% reduction in Y-BOCS
Hazari et al., 2016 [39]	Case report One 24 year-old-male	Anode: left pre-SMA; Cathode: right supraorbital area 2 mA, 20 min twice a day for 10 days	• 80% reduction in Y-BOCS
Dinn et al., 2016 [40]	Open label trial Four females and one male	Anode: F3; Cathode: Fp2 2 mA, 20 min daily for 15 days	• 23% reduction in Y-BOCS but was not maintained at 1 month of follow-up • No reduction in anxiety symptoms and 30% reduction in depression symptoms
Najafi et al., 2017 [41]	Open label trial Twenty three females and nineteen males	Anode: P1, C3, T7; Cathode: Fp2 2 mA, 30 min daily with total 15 stimulations in 3 weeks	• 79% reduction in Y-BOCS
Bation et al., 2016 [20]	Open label trial Six females and two males	Anode: right cerebellum; Cathode: left OFC 2 mA, 20 min twice a day for 5 days	• 26% reduction in Y-OBCS
D’Urso et al., 2016 [21]	RCT Seven females and four males	Active: Cz; Reference: lateral surface of right deltoid 2 mA, 20 min daily for 10 days	• Cases with anodal stimulation (*N* = 6) had worsening in Y-BOCS • Cases with cathodal stimulation (N = 6) had 20% reduction in Y-BOCS
Yekta et al., 2015 [42]	Controlled RCT Twenty cases (no data on gender)	Anode: F4; Cathode: F3 2 mA; 20 min daily for 15 days in experimental group	• Improved decision-making of experimental group (*N* = 10) compared to sham control group (N = 10) • No report on Y-BOCS
Gowda et al., 2019 [43];	Controlled RCT Four females and 21 males	Anode: pre-SMA; Cathode: right supraorbital area 2 mA, 20 min twice a day for 5 days	• Y-BOCS was significantly reduced in experimental group compared to baseline and sham control arm
Bation et al., 2020 [44];	Controlled RCT	Anode: right cerebellum; Cathode: PF1 2 mA, 20 min twice a day for 5 days	• Significant acute effect of tDCS in experimental group compared to baseline and sham control arm • No changes in experimental group was observed compared to sham control arm after 1 and 3 months of tDCS stimulation

Four randomized clinical trials assessed the efficacy of tDCS on people with OCD (Table 3) [21, 42–44]. A randomized double-blind sham controlled study suggested that tDCS could be effective in people with moderate to severe OCD (Y-BOCS> 16) who were resistant to SSRI therapy [43]. Another recent RCT with sham controlled arm showed the significant acute effect of tDCS on individuals with OCD compared with control arm but did not find any statistical difference between groups in 1 and 3 months after treatment [44]. Discrepancies in results of these trials can be due to the methodological differences and small sample sizes. The cathodal and anodal placements in these studies were different to each other and to the present study. Furthermore, none of the above-mentioned trials assessed the efficacy of tDCS as add-on therapy.

### Strengths, limitations, and future directions

Randomization, blinding, and the presence of sham controlled arm were the major strengths of this study. The prospective population-based approach was another important strength of our trial that can increase the external validity of the reported results. Small sample size and short periods of follow-up were main limitations of trial. OC symptoms cannot be objectively evaluated and our outcomes were reliant on the reports of individuals using Y-BOCS test. This can lead to measurement bias (hawthorne effect). The cathodal stimulation with tDCS on individuals with OCD was shown to be effective in case-report studies more than anodal stimulation. Future controlled RCTs should investigate this hypothesis. Further studies with higher number of cases should also evaluate the efficacy of tDCS as the only therapy on improvements of OC symptoms in cases with different severity.

## Conclusions

The tDCS adjunctive therapy with the anode placed over left DLPFC and cathode placed over right OFC was generally safe and a tolerable procedure. This study didn’t show significant clinical efficacy of add-on therapy with tDCS in the management of individuals with moderate to severe OCD compared to the control group.

## Data Availability

The datasets generated and/or analysed during the current study are not publicly available but are available from the corresponding author on reasonable request.

## Abbreviations

OCD: Obsessive-Compulsive Disorder
tDCS: transcranial Direct Current Stimulation
Y-BOCS: Yale-Brown Obsessive-Compulsive Scale
SSRIs: Selective Serotonin Reuptake Inhibitors
CBT: Cognitive-Behavioral Therapy
DLPFC: Dorsolateral Prefrontal Cortex
OFC: Orbitofrontal Cortex
SMA: Supplementary Motor Area
DSM-5: Diagnostic and Statistical Manual of Mental Disorders
ECT: Electroconvulsive Therapy
RCT: Randomized clinical trial
